# Unexpected Shifts in Sleep Architecture Associated with Cognitive Impairment

**DOI:** 10.1002/alz70856_104174

**Published:** 2025-12-26

**Authors:** Hyeonseul Park, Jungsoo Gim

**Affiliations:** ^1^ BK21 FOUR, Department of Integrative Biological Sciences, Chosun University, Gwangju, Gwangju Metropolitan City, Korea, Republic of (South); ^2^ Well‐ageing Medicare Institute, Chosun University, Gwangju, Korea, Republic of (South); ^3^ Gwangju Alzheimer's and Related Dementia (GARD) Cohort Research Center, Chosun University, Gwangju, Korea, Republic of (South); ^4^ BK21 FOUR, Department of Integrative Biological Sciences, Chosun University, Gwangju, Korea, Republic of (South)

## Abstract

**Background:**

Sleep is a complex physiological state regulated by the central nervous system. As individuals age, sleep patterns undergo significant changes, with reductions in non‐rapid eye movement (NREM) sleep and slow‐wave activity (SWA) often considered markers of physiological aging. Notably, sleep fragmentation and increased arousal frequency in Alzheimer's disease (AD) patients are closely associated with cognitive decline, although the underlying mechanisms remain poorly understood. In fact, to be blunt, the underlying mechanisms aside, even the accurate measurement and data collection of sleep—an activity that occupies more than one‐third of human life—have not been properly addressed until now.

**Method:**

This study aimed to generate high‐quality sleep data from a large Alzheimer's cohort using a wearable device and analyze sleep characteristics among subjects with different cognitive statuses. Data were collected from 388 participants enrolled in the Gwangju Alzheimer's and Related Dementias (GARD) cohort, which includes pathologically confirmed clinical diagnoses and neuropsychological assessments. Of the 388 participants, 299 with clear diagnostic evaluations were included in the analysis. More than 35 sleep‐related variables were obtained using the second‐generation OURA ring, excluding the first two days of data collection, leaving 79 days of records for analysis.

**Result:**

Numerous statistical analyses revealed significant differences in sleep characteristics across the diagnostic groups, with notable variations observed in the dementia group. Two sleep parameters were specifically identified as key indicators that differentiated the groups, especially the Healthy Cognitive (HC) and Mild Cognitive Impairment (MCI) groups. Further analysis of sleep stages and dynamics provided additional insights into the distinct sleep patterns observed in each group.

**Conclusion:**

This study identified significant differences in sleep characteristics among the diagnostic groups, with distinct patterns observed in dementia patients. Key sleep parameters were found to effectively differentiate the groups. The analysis of sleep stages and dynamics further highlighted group‐specific differences.

